# Freiburg Neuropathology Case Conference: An 89-year-old Patient with a History of Domestic Falls, Dysarthria and a slowly Progressive Cerebellar Mass Lesion

**DOI:** 10.1007/s00062-022-01142-5

**Published:** 2022-02-23

**Authors:** S. Rau, M. Frosch, M. J. Shah, M. Prinz, H. Urbach, D. Erny, C. A. Taschner

**Affiliations:** 1 Departments of Neuroradiology, University of Freiburg, Freiburg, Germany; 2 Neuropathology, University of Freiburg, Freiburg, Germany; 3 Neurosurgery, University of Freiburg, Freiburg, Germany; 4 Medical Centre—University of Freiburg, Faculty of Medicine, University of Freiburg, Breisacherstr. 64, 79106 Freiburg, Germany

**Keywords:** Hemangioblastoma, Solitary fibrous tumor of the dura, Meningioma, Brain metastases, Radiologic-pathologic correlation

## Case Report

**Fig. 1:**
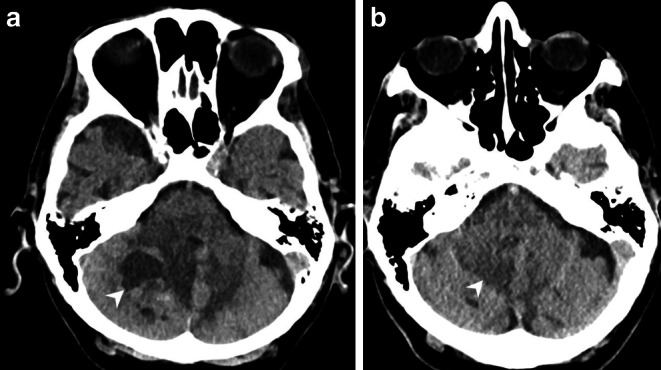
Axial cranial CT images at admission (**a**) in soft tissue window showed a well-circumscribed mass lesion located in the right cerebellar hemisphere (*arrowhead*). The patient had another cranial CT 3.5 years prior to admission (**b**). In retrospect, the lesion was already detectable (*arrowhead*) but appeared much smaller

**Fig. 2:**
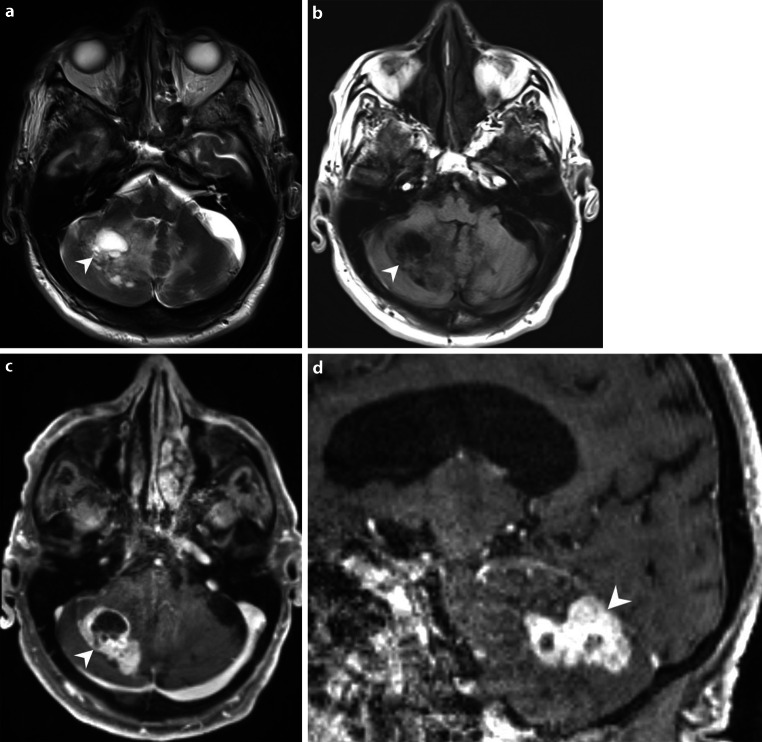
Axial T2 weighted images (**a**) showed a space-occupying lesion (*arrowhead*) composed of cystic as well as solid components, surrounded by a perifocal oedema. On axial native T1 weighted images (**b**) the lesion (*arrowhead*) appeared hypointense when compared to the cerebellar tissue. On axial (**c**) and sagittal (**d**) T1 weighted images after administration of gadolinium the solid portions of the lesion show marked and homogeneous contrast enhancement(**c**, *arrowhead*). The lesion displayed extended contact to the tentorium without any linear meningeal thickening or adjacent enhancement (**d**, *arrowhead*)

**Fig. 3:**
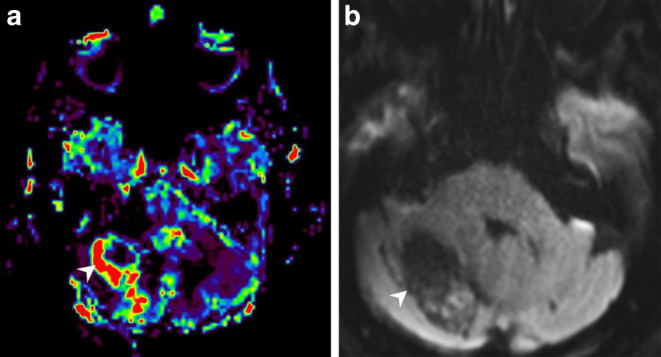
Corresponding relative cerebral blood volume (rCBV) maps (**a**) show increased perfusion (*arrowhead*) of the nodular portions of the lesion compared to normal brain tissue. On diffusion weighted images with a b‑value = 1000 (**b**), the lesion did not show any signs of restricted diffusion (*arrowhead*)

An 89-year-old patient was admitted through our Accident and Emergency department after a domestic fall. Upon neurological examination, the patient appeared somnolent and had a dysarthric speech. A cranial computer tomography (CT, Fig. 1a), as well as subsequent magnetic resonance imaging (MRI, Figs. 2 and 3) of the head revealed a right cerebellar mass. A cranial CT also performed in relation with a domestic fall 3.5 years earlier already showed a small hypodense lesion in the same location (Fig. 1b). Due to the increase in size and the increasing mass effect of the lesion, with compromised cerebrospinal fluid (CSF) outflow, surgery was recommended. The operation was performed with the patient under general anesthesia and in a prone position. After suboccipital craniotomy, access to the tumor was gained. The tumor was found to be hard and very bloody and was removed circumferentially. Despite its proximity to the tentorium, the tumor was located strictly intra-axially.

The patient was extubated on the first postoperative day without any new focal neurological deficit; however, mobilisation was difficult and the patient was only discharged from the intensive care unit on the seventh postoperative day. The patient unexpectedly succumbed 3 days later, most likely due to a pulmonary embolism.

## Imaging

The cranial CT upon admission (Fig. 1a) revealed a well-circumscribed right cerebellar mass. In retrospect, the lesion had already been apparent on a previous cranial CT performed 3.5 years earlier. At that time the lesion appeared to be much smaller (Fig. 1b). On T2 weighted images from the current MRI (Fig. 2a) the lesion had a multicystic lobulated matrix and presented with a space-occupying effect and surrounding hyperintense signal alterations in fluid attenuated inversion recovery images (FLAIR, not shown) extending to the contralateral side. The local mass effect included a displacement of the fourth ventricle and consecutive signs of an obstructive hydrocephalus with enlargement of the lateral ventricles and the third ventricle and a periventricular oozing (not shown). On native T1 weighted images (Fig. 2b) the lesion was hypointense. On T1 weighted images after administration of gadolinium the walls of the cystic components as well as the nodular parts of the lesion showed homogeneous and intense contrast enhancement (Fig. 2c). The lesion had a broad-based contact to the inconspicuously configured tentorium cerebelli (Fig. 2d). The nodular parts of the mass showed signs of high perfusion and hypervascularisation in the MRI-perfusion relative cerebral blood volume (rCBV) map compared to normal brain tissue (Fig. 3a). On diffusion weighted images (b‑value = 1000, Fig. 3b), the lesion did not show any signs of restricted diffusion.

## Differential Diagnosis

### Hemangioblastoma

Hemangioblastomas are benign (WHO grade I), slow growing, vascular and relatively rare (7%) neoplasms of the posterior fossa. Second to metastasis, they are the most common posterior fossa tumor in adults [1]. Even though large case series reported an age peak for hemangioblastomas between 30 and 65 years old, the prevalence for hemangioblastomas in patients > 65 years old has been reported with up to 13.6% [2]. Hemangioblastomas occur in both sporadic and multiple forms, whereas the multiple form is associated with von Hippel-Lindau (VHL) disease [3]. In VHL hemangioblastomas are usually located in the posterior fossa (60–76%) and four different morphological types of hemangioblastomas have been described: solid (48%), cystic (26%), cystic with mural nodules (21%), and both cystic and solid (5%) [1]. The clinical presentation largely depends on the degree of mass effect with a long history (6–10 months) of minor symptoms followed by a sudden exacerbation due to high intracranial pressure (50% of presentations), most often related to cerebrospinal fluid (CSF) obstruction [1, 4]. Radiological features include a well-circumscribed hypointense to isointense T1-weighted and hyperintense T2-weighted mass, with intense contrast enhancement of the nodular parts as well as vascular flow voids in the surrounding tissue. The cysts contain fluid slightly hyperintense to CSF fluid in T1-weighted images without a contrast-enhancing wall [5, pp. 606–609].

In the present case, we considered hemangioblastoma to be a valid differential diagnosis. The radiological features of the cerebellar mass matched with many of the described patterns especially the hypervascularisation and the cystic components of the lesion. Furthermore, the slow size progression in the past 3.5 years was in line with the diagnosis of a nonmalignant tumor.

### Solitary Fibrous Tumor of the Dura

The solitary fibrous tumor of the dura replaces the tumor entity previously referred to as hemangiopericytoma since the 5th edition of the WHO classification of CNS tumors from 2021 [6]. With only about 0.4% of all CNS tumors it is very rare and mainly occurs around the age of 40 years (20–65 years). Depending on the subtype and the tumor size the symptoms can vary; most commonly headaches followed by seizures, visual dysfunction and motor weakness have been reported [7]. Solitary fibrous tumors of the dura are thought to originate from mesenchymal spindle cells and are located mostly along the occipital dura, originating from the falx or tentorium cerebelli with or without a dural tail sign. They often show signs of hypervascularisation including prominent flow voids [5, p. 605]. In a case series by Zhou et al. 39 patients with anaplastic hemangiopericytomas (former WHO grade III) were analysed by their MRI appearance. The image findings included lobulations/cross-leaf growth, necrosis and cystic changes, a rare dural tail sign, bleedings, more significant oedema and damage of the nearby skull as well as extracranial metastases [8].

In our patient some of the imaging features of solitary fibrous tumors of the dura, more precisely of the former subtype anaplastic hemangiopericytoma were present. Nevertheless, the lesion showed no sign of osseous infiltration or metastases and had a slow growth progression. This differential diagnosis had to be considered, yet it seemed less likely due to the lack of dural involvement and the advanced age of the patient.

### Meningioma

Meningiomas account for about 20% of all intracranial tumors with a peak incidence at 45–55 years old [9]. They are most commonly located supratentorially but are also found in approximately 9–15% of patients in the posterior fossa [10]. Usually, they demonstrate as an extra-axial, well-circumscribed, contrast enhancing (in about 90%) mass with broad-based dural attachement and CSF cleft. In nonenhanced cranial CT they are mostly hyperdense (70%) to isodense (30%) and in 25% with homogeneous, sand-like or sprinkled calcifications. Additionally, hyperostotic or permeative sclerotic bone changes are possible. In non-enhanced MRI they have an isointense to minimally hyperintense signal in T1w and variable signal in T2w images and are often surrounded by a perifocal oedema (60%). Within highly vascular meningiomas T2-flow voids are seen. The typical dural tail sign can be found but is not specific. Furthermore, cysts (2–4%), necrosis and hemorrhage are also possible but uncommon features [11]. Atypical (WHO grade II) and anaplastic/malignant (WHO grade III) meningiomas tend to be more aggressive and account for about 10% of meningiomas. Unlike WHO grade I meningiomas, they are indistinct from or infiltrate the brain parenchyma. Image morphological differentiation of the meningioma-types is hardly possible, but a large perifocal oedema and a low apparent diffusion coefficient (ADC) indicates high-grade variants [11].

In our patient, the mass showed a small contact zone with the tentorium but neither a dural tail nor osseous involvement was present. Furthermore, the lesion seemed to be located intra-axially, which, together with the perifocal oedema, would point in the direction of an atypical or anaplastic meningioma (WHO grade II/III). Considering the clinical course and the morphology of the underlying lesion, the diagnosis of a meningioma seemed possible but not very likely.

### Brain Metastases

Brain metastases occur in 20–40% of systemic tumor diseases with a peak prevalence at 65 years and older. They are less commonly located in the cerebellum (15%) and the brainstem but still account for the most common malignancies of the posterior fossa (around 75%) in adults [12, 13]. Most commonly, the primary malignancies in infratentorial metastases are lung and breast cancers. In melanomas, posterior fossa metastases are very rare [14, 15]. Brain metastases can be asymptomatic or lead to a variety of neurological symptoms especially seizures and local mass effect-induced symptoms [16]. The imaging features of brain metastasis are quite variable. Most commonly, they present as oval nodular or ring-enhancing lesions with a perilesional oedema. They can also contain central necrotic/cystic portions [5, p. 755]. In perfusion imaging, they often present with elevated rCBV compared to normal brain tissue. Even though several entities such as renal cell carcinoma or melanoma show hypervascular metastases, pronounced flow voids are uncommon in brain metastasis [17]. The slow growth progression of the cerebellar lesion over the course of 3.5 years made this diagnosis highly unlikely in our patient.

## Histology and Immunohistochemistry

**Fig. 4:**
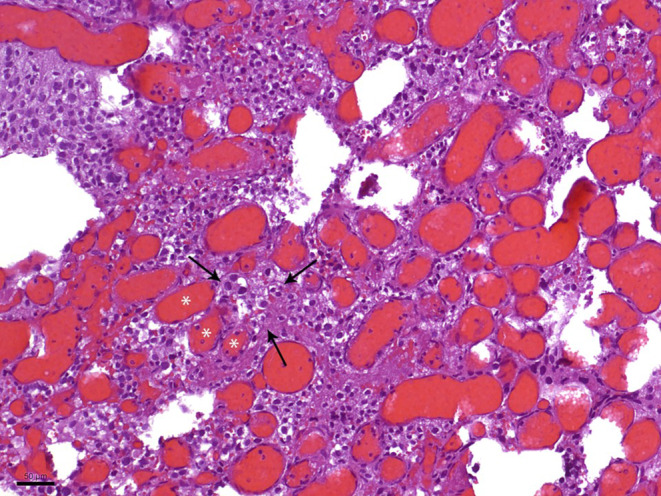
Hematoxylin and eosin (H&E) stain showed a tumor with two major components. The neoplastic stromal cells were partially vacuolated. The vascular cells appeared more abundant. *Asterisks* indicate vascular lumen, *arrows* the neoplastic stromal component. Scale bar: 50 µm

**Fig. 5:**
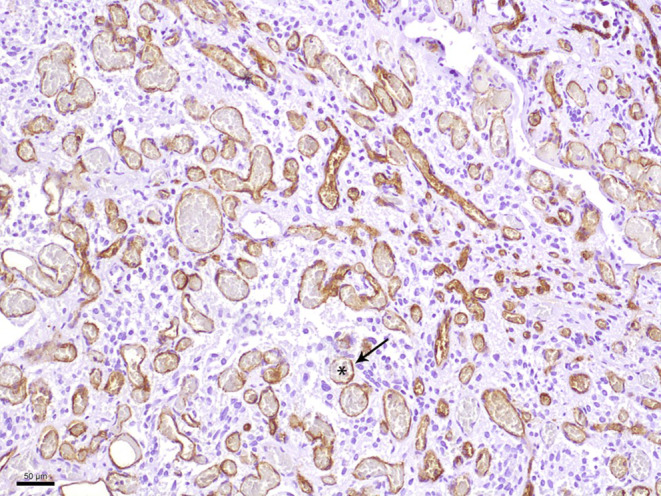
CD34 immunoreactivity was only present in the endothelium of the numerous vessels but not in the stromal cells. *Asterisk* indicates vascular lumen. The *arrow* points to the CD34-positive endothelial layer. Scale bar: 50 µm

**Fig. 6:**
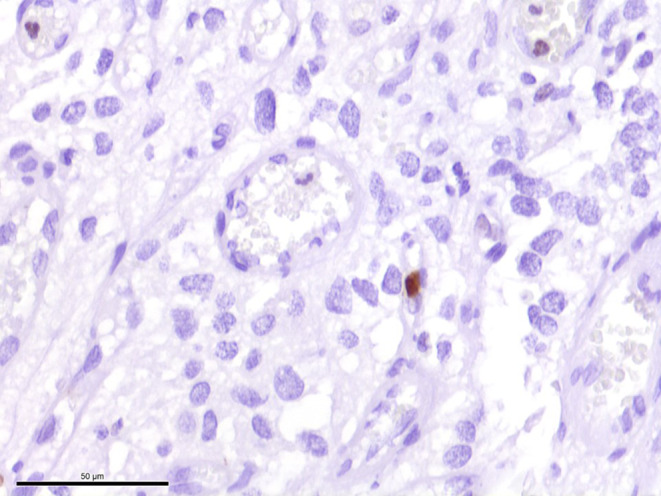
Staining against the proliferation marker Ki-67 showed only isolated positive cells. Scale bar: 50 µm

In the hematoxylin and eosin (H&E) stained sections of the formaldehyde-fixed and paraffin-embedded biopsy material, fragments of a highly vascular tumor were found (Fig. 4). The vascular cells within the tumor are more abundant than the neoplastic stromal cells. The immunohistochemical reaction against CD34 marks the endothelial layer of these vessels but not the stromal cells (Fig. 5). Most vessels are small in diameter and are best termed “capillary”. In addition, larger vessels appear within the tumor too. The stromal tumor cells lying between the capillaries often exhibit a roundish nucleus of moderate chromatin density. Only a few tumor cells show a hyperchromatic, atypical nucleus. Mitotic figures are scarce within the tumor cells, in line with a low proliferative activity of less than 1%, as shown in the immunohistochemical staining against Ki-67 (Fig. 6).

**Fig. 7:**
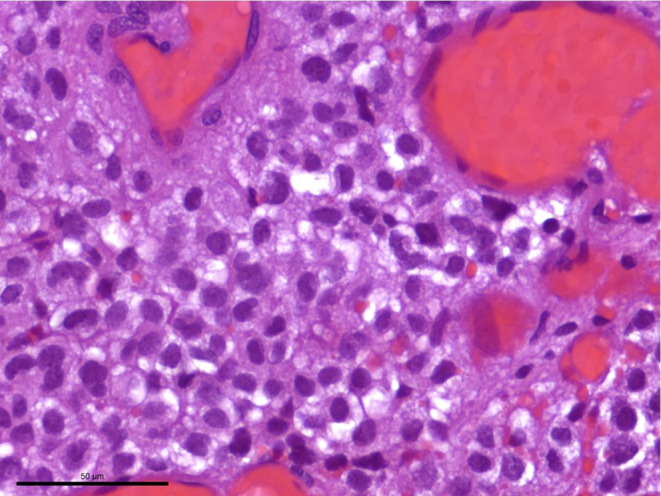
Many tumor cells exhibited a characteristic multi-vacuolated cytoplasm. Scale bar: 50 µm

**Fig. 8:**
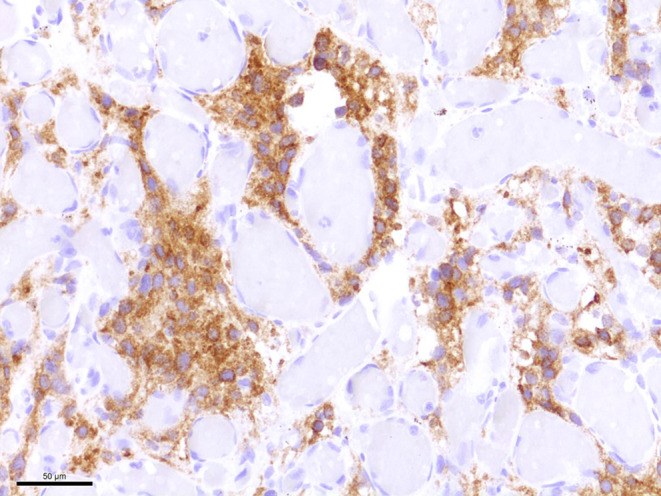
Neoplastic stroma cells were strongly stained in the immunohistochemical reaction for inhibin alpha. Scale bar: 50 µm

**Fig. 9:**
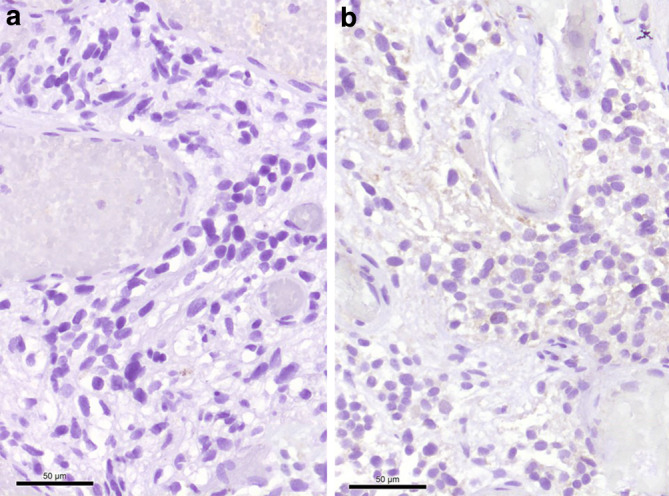
Immunohistochemical reactions against EMA (**a**) and STAT6 (**b**) remained negative. Scale bar: 50 µm

Moreover, many tumor cells exhibit medium to large cell bodies with multiple vacuoles (Fig. 7). A smaller portion of the tumor cells has a slightly epithelioid appearanceand grows in a more solid pattern. Many smaller and fresh hemorrhages can be multifocally observed. Hemosiderin deposits as a sign of older bleedings are not seen. Most tumor cells show a strong signal in the immunohistochemical reaction for inhibin alpha (Fig. 8). Immunohistochemical reactions against the epithelial membrane antigen (EMA, Fig. 9a) and STAT6 remain negative (Fig. 9b). Furthermore, gliotic altered cerebellar brain tissue is found in the border regions of the biopsy. This brain tissue appears sharply demarcated from the adjacent tumor tissue. In summary, the histopathological finding of a tumor with two major components, namely neoplastic stromal cells that appear partially vacuolated and abundant vascular cellularity, leads to the diagnosis of a hemangioblastoma, CNS WHO grade I.

## Diagnosis

### Hemangioblastoma (WHO Grade I)

Hemangioblastomas are rare benign neoplasms that account for less than 2% of all intracranial tumors. They typically occur in the cerebellum (up to 76%; like in the outlined case) and are less frequent in the brainstem or along the spinal cord [1, 18]. Most hemangioblastoma cases occur sporadically or less commonly associated with VHL syndrome [19]; however, about 70–80% of VHL patients exhibit hemangioblastomas of the central nervous system (CNS). VHL-associated hemangioblastomas tend to appear at a younger age than sporadic forms (30–40 years vs. 50–70 years), but both are primarily seen in adults [20]. In the described case, VHL was not known, and the patient’s age was 89 years which is older than the age-related peak incidence but still not uncommon for sporadic cases [19].

The differential diagnosis for highly vascular tumors with a low proliferation rate includes a solitary fibrous tumor (SFT; formerly known as hemangiopericytoma), CNS WHO grade 1, as well as an angiomatous meningioma, CNS WHO grade I [6]. The SFTs are also rare tumors within the CNS that make up less than 1% of all CNS tumors. They are usually found supratentorial, superficial, and closely related to the meninges [21]; however, there are rare cases reported with a cerebellopontine localization of SFTs [22]. Peak incidence occurs between 50 and 70 years [23, 24]. On the histopathological level, SFTs are characterized by prominent, branching, and staghorn-shaped blood vessels and randomly arranged spindled-ovoid monomorphic cells between these vessels. Molecularly, SFTs show a genomic inversion at the 12q13 locus leading to a NAB2:STAT6 fusion. This fusion conditions a solid nuclear expression of STAT6, which can be detected and seen as an immunohistochemical hallmark of SFTs [25]. In the above-explained case, the missing nuclear STAT6 expression and the cerebellar localization of the tumor make the diagnosis of an SFT unlikely.

Angiomatous meningiomas are a meningioma variant graded as CNS WHO grade 1. Meningiomas per se are a frequent entity of brain tumors (37.6% of all CNS tumors) occurring most likely in older patients. The risk increases with age [26]. In the angiomatous variant, numerous blood vessels often represent a greater portion than the meningioma cells themselves [6]. The blood vessels are often thick-walled and hyalinized. The tumor cells exhibit a positive signal in the immunohistochemical reaction for EMA. EMA immunohistochemistry can help identify the occasionally sparse tumor cells between the blood vessels and exclude differential diagnoses, such as hemangioblastoma, where tumor cells are negative for EMA.

In the current case, clinicians also thought about the clinical presentation of a brain metastasis. Indeed, histologically, hemangioblastomas can present hypercellular, and the vacuolation of the tumor cells can impress as a clear cell component that resembles metastatic renal clear cell carcinoma [6]. In such cases, immunohistochemistry for renal clear cell carcinoma markers such as renal cell carcinoma marker (RCCm) or CD10 may help differentiate these entities. In addition, the fact that patients with VHL syndrome tend to develop renal cell carcinomas can make it necessary to exclude a cerebral metastasis with the mentioned immunohistochemistry [27].
